# Roles of micrornas in the Hepatitis B Virus Infection and Related Diseases

**DOI:** 10.3390/v5112690

**Published:** 2013-11-07

**Authors:** Muriel Thirion, Takahiro Ochiya

**Affiliations:** Division of Molecular and Cellular Medicine, National Cancer Center Research Institute, 5-1-1 Tsukiji, Chuo-ku, Tokyo 104-0045, Japan; E-Mail: mthirion@ncc.go.jp

**Keywords:** hepatitis B virus, microRNA, hepatocellular carcinoma

## Abstract

The hepatitis B virus (HBV) is a small enveloped DNA virus that belongs to the *Hepadnaviridae* family. HBV can cause acute and persistent infection which can lead to hepatocellular carcinoma (HCC). MicroRNAs (miRNAs) play a crucial role in the main cellular events. The dysregulation of their expression has been linked to the development of the cancer as well as to viral interference. This chapter will describe the involvement of miRNAs in the case of HBV infection and their implication in the development of the HBV-related diseases.

## 1. Introduction

The microRNAs (miRNAs or miRs) are small non-coding RNAs of 19–23 nucleotides that play key roles in the regulation of almost every cellular process in all multicellular eukaryotes [1]. As intracellular pathogens, viruses are affected by these post-transcriptional modulators and have evolved to subvert them. Several viruses, especially the herpesviruses, encode for their own miRNAs that increase their replication potential and/or allow the evasion from the innate immune system [2]. Other viruses, such as the hepatitis B virus (HBV), modulate the cellular miRNAs in order to achieve the same effects. 

HBV is a small enveloped DNA virus that belongs to the *Hepadnaviridae* family. It primarily infects hepatocytes and causes acute and chronic liver disease. Among the 2,000 million people worldwide infected with HBV, more than 350 million remain chronically infected and become carriers of the virus [3]. Epidemiological studies have uncovered chronic HBV infection as the major etiological factor in the development of hepatocellular carcinoma (HCC) [4]. Despite the availability of an efficient vaccine, persistent HBV infection remains a challenging global health issue. The recent discovery of miRNAs involvement in HBV infection provides new insights into the virus biology and pathogenesis [5,6].

This chapter will outline the roles of miRNAs in the HBV biology and associated pathogenesis. We will also outline present and future miRNA-based strategies for the diagnosis, prognosis and treatment of the HBV-related diseases.

## 2. Biogenesis and Functions of miRNAs

**Figure 1:**
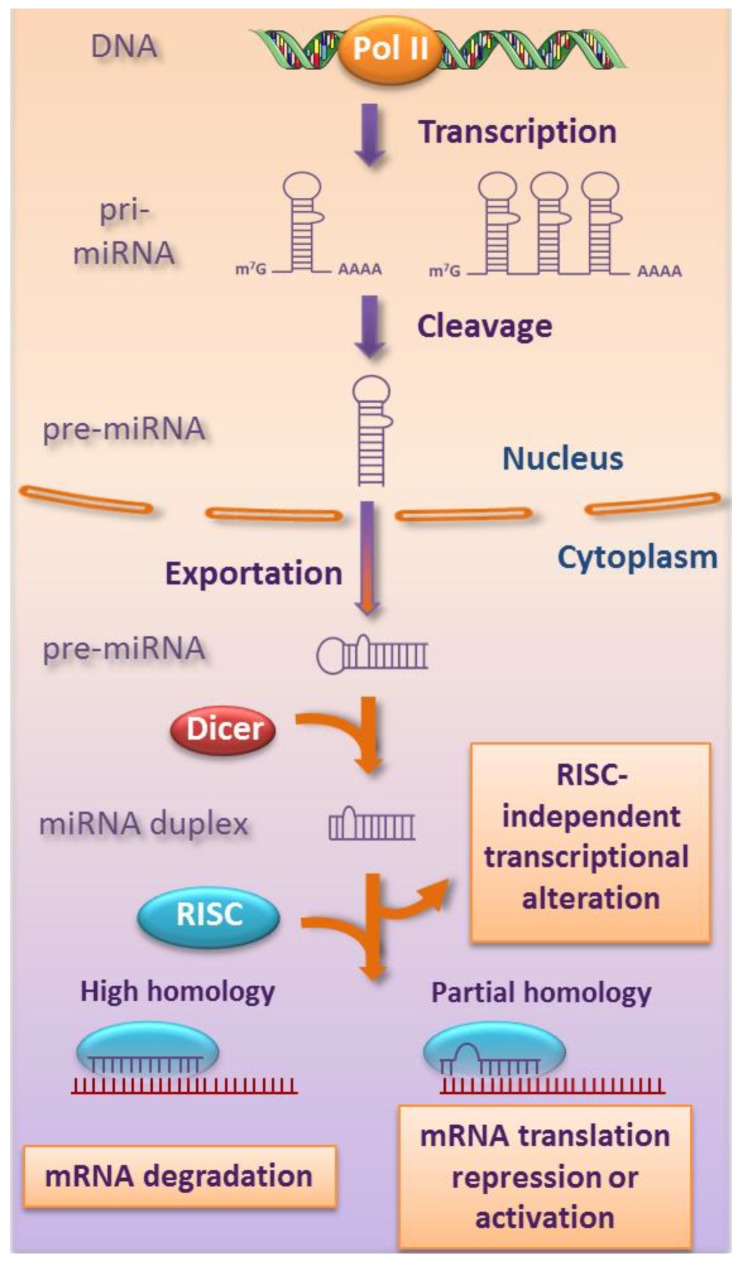
Schematic representation of miRNA biogenesis. The mature miRNAs originate from successive different steps. An initial DNA transcription generates pri-miRNAs that are cleaved in pre-miRNAs before their exportation to the cytoplasm. There, an RNAse III enzyme, Dicer, cleaves it to generate a miRNA duplex that subsequently joins an RNA‑induced silencing complex (RISC) to produce the mature miRNA. The sequence complementarity to the target will decide its fate. Some miRNAs can act on the transcriptional level independently from the RISC.

miRNAs are most commonly transcribed in the nucleus by the RNA polymerase II (Pol II), as monocistronic or polycistronic pri-miRNAs that are further processed in pre-miRNAs (Figure 1). These pre-miRNAs are exported to the cytoplasm where they undergo cleavage by the RNAse III enzyme called Dicer that produces a miRNA duplex. This duplex splits to generate the single-stranded mature miRNA that incorporates the RNA-induced silencing complex (RISC). Based on the complementarity with its target gene sequence, the mature miRNA induce either translational repression (partial complementarity) or mRNA degradation (perfect complementarity) [7]. Besides, the mature miRNA can increase the expression of the target gene under growth arrest condition [8]. Finally, it has been recently reported that miRNA can also act in a RISC-independent manner on the transcriptional level by interaction with ribonucleoprotein or direct binding to DNA [9,10,11]. 

One single miRNA has the ability to regulate multiple targets and thereby to affect a broad network of genes (up to 100 genes) [12]. This specific characteristic makes the miRNAs key mediators of most of the cellular events. In animal, miRNAs mainly regulate mRNAs by interacting with their 5' end (5p) to the 3'-untranslated region (3'-UTR) of their target [13]. However, recent studies have revealed miRNAs target sites in the 5'-UTR, which interacts with the 3' end (3p) of miRNAs, and even simultaneous 5'-UTR and 3'-UTR interaction sites [14,15]. 

The abnormal expression levels of miRNAs have been revealed in various diseases such as cancer [16,17], inflammation [18,19], Alzheimer [20], cardiovascular disease [21] and viral infection including HBV [2,22].

## 3. Role of miRNAs in HBV Infection

Despite the fact that HBV is a nuclear DNA virus, none viral-encoded miRNA has been so far identified. Only one putative HBV miRNA, with hypothetical regulation role on its own genome, was deduced by computational approach [23]. However, HBV can modulate the expression of several cellular miRNAs in order to promote a favorable environment for its replication and survival. They are presented in this section and summarized in Table 1.

### 3.1. Brief Description of HBV Infection

**Figure 2:**
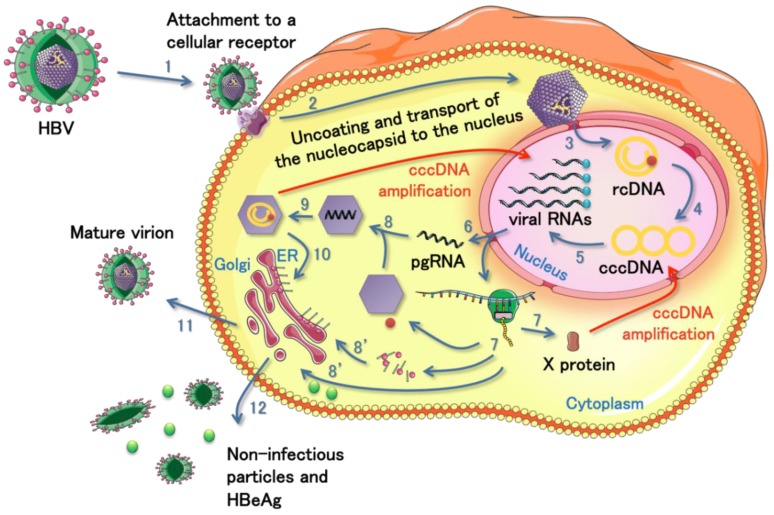
Schematic representation of HBV life cycle. The virus infects a cell by an initial attachment to a cellular receptor that allows its internalization (step 1). In the cytoplasm, the virus is uncoated and the nucleocapsid is transported to the nuclear membrane (step 2). The viral genome is released into the nucleus under its relaxed circular form (rcDNA) and converted into a covalently closed circular DNA (cccDNA) from which all the viral RNAs are produced (steps 3 to 5). The viral RNAs transfer to the cytoplasm for traduction of the different viral proteins (steps 6 and 7) or for subsequent reverse transcription of the pregenomic RNA (pgRNA, steps 6, 8 and 9). All the viral components move to the proper place and assemble together to form new mature virions (steps 8, 10 and 11). The virus also produces non-infectious particles and extracellular antigen (HBeAg) as a decoy for the immune system of the host (step 12). The nucleocapsid containing the rcDNA and the HBV X protein (HBx) can go back to the nucleus in order to amplify the cccDNA and maintain the viral production.

The HBV infection is characterized by two phases; the acute and the chronic infection [24]. The initial stages of the acute infection include virion attachment [25], uncoating and nucleocapsid transport to the cell nucleus (Figure 2, steps 1 and 2). The 3.2 kb relaxed circular DNA genome is released into the nucleus and converted into a covalently closed circular DNA (cccDNA) from which all the viral RNAs are transcribed (Figure 2, steps 3 to 5). The pregenomic RNA (pgRNA) serves as template for reverse transcription (Figure 2, steps 8 and 9). The subgenomic mRNAs comprise the pre-surface (S) and S genes, the pre-core (C) and C genes, the polymerase gene, and the X gene. The newly formed nucleocapsids can either assemble with envelope proteins in the endoplasmic reticulum and form mature virions that will be secreted (Figure 2, steps 10 and 11), or return to the nucleus to maintain the cccDNA amplification. When the immune system fails to clear the virus, the HBV infection becomes chronic and it remains under a dormant state into the cell. Eventually, the viral genetic material or sequences can integrate into the host cellular DNA. The integration has been frequently observed and is associated with HCC [26,27].

**Table 1:** Cellular miRNAs and their effects on HBV infection or HBV related-diseases. HBV (↑): Promotes HBV replication; HBV (↓): Inhibits HBV replication; HCC (↑): Development and/or growth of HCC; Fibrosis (↑): Promotes liver fibrosis.

miRNAs	miRNA expression	Target genes	HBV or disease status	Ref.
**Cellular targets**				
*miR-1*	up	HDAC4 (histone deacetylase 4)	HBV (↑)	[28]
*miR-17-92 cluster*	up	E2F1 (c-myc repressor)	HBV (↑?), HCC (↑)	[29]
*miR-155*	up	C/EBPβ (CCAAT/enhancer binding protein)	HCC (↑)	[30]
		SOCS1 (JAK/STAT signaling)	HBV (↓)	[31]
*miR-181a*	up	HLA-A? (MHC class I)	HBV (↑)	[5]
*miR-372*	up	NFIB (nuclear factor I/B)	HBV (↑)	[32]
*miR-373*	up	NFIB (nuclear factor I/B)	HBV (↑)	[32]
*miR-501*	up	HBIP (HBx inhibitor)	HBV (↑)	[33]
*mir-29 family*	down	collagen	Fibrosis (↑)	[22,34]
*miR-122*	down	cyclin G1 (p53 modulator)	HBV (↑), HCC (↑)	[6]
*miR-152*	down	DNMT1 (DNA methyltransferase 1)	HBV (↓)	[35]
*let-7 family*	down	STAT3 (transcription factor)	HBV (↑?), HCC (↑)	[36]
**Viral targets**				
*miR-122*	up	HBV DNA polymerase	HBV (↓)	[37]
*miR-125a-5p*	up	HBsAg (HBV surface antigen)	HBV (↓)	[38]
*miR-199-3p*	up	HBsAg	HBV (↓)	[39]
*miR-210*	up	HBV pre-S1 (pre-surface 1)	HBV (↓)	[39]

### 3.2. Role of miRNAs in the HBV Replication

The role of miRNAs in HBV replication is therefore dependent on the phase of HBV infection. During the acute phase, the virus must activate its replication while avoiding destruction by the immune system. During the chronic phase, the virus reaches a “dormant” state where the viral replication must be restricted and viral evasion maintained. This leads to a time-dependent intricate interaction network in which miRNAs play an important role.

One of the best studied miRNAs in HBV infection and other liver-related diseases is miR-122. This liver-specific miRNA is expressed at high levels in normal hepatocytes (about 70% of the total miRNA population in the adult liver) [40] and is pivotal in numerous aspects of the liver function such as lipid metabolism, liver development, differentiation, growth and neoplastic transformation [41]. While the loss of miR-122 expression impedes hepatitis C virus (HCV) replication [42], it enhances the replication in the circumstance of HBV infection [6]. However, miR-122 can negatively regulate the viral gene expression and replication by direct binding to a highly conserved sequence of HBV [37]. This repression effect can apparently be impeded by a negative feedback loop involving the Heme oxygenase-1 [43]. A recent study has reported the indirect implication of the HBV X protein (HBx) in miR-122 dysregulation [44] that could, at least partially, explain the difference observed between the two viruses.

On the other hand, miR-1 can enhance the HBV core promoter transcription by down-regulating the expression of the histone deacetylase 4 (HDAC4) [45]. This miRNA might act complementary to the nuclear HBx in order to induce epigenetic modifications on the cccDNA and amplify the viral genome [45,46]. 

miR-372, together with miR-373, also supports HBV gene expression by targeting the nuclear factor I/B [47]. This cellular protein is known to be an important regulator of several viruses [48].

The let-7 family of miRNAs has been demonstrated to be negatively regulated by HBx [36]. The consequence of this down-regulation is the increase activity of the signal transducer and activator of transcription 3 (STAT3) that supports cell proliferation, and potentially viral replication and hepatocarcinogenesis. 

Finally, miR-501 has been suggested to work with HBx for the benefit of viral replication [33]. HBx itself has also the ability to dysregulate the cellular miRNAs expression. This small protein is a key regulator of HBV infection. It is usually overexpressed in HCC and is involved in hepatocarcinogenesis [48].

### 3.3. Role of miRNAs in the Immune Evasion of HBV

miRNAs are important in the development and function of immune system [49]. In particular, miR-155 has multi-roles during innate immune response such as regulation of the acute inflammatory response after recognition of pathogens by the toll-like receptors [50,51]. Su and collaborators demonstrated that ectopic expression of miR-155 in human hepatoma cells could enhance the innate immunity through promotion of the janus kinase (JAK)/STAT pathway and down-regulate HBx expression [31].

On the other hand, a study analyzing the modified expression profiles of miRNAs in a stable HBV-expressing cell line revealed the upregulation of miR-181a [5]. The dysregulation of this miRNA in liver cell might participate to HBV replication through inhibition of the human leukocyte antigen A (HLA-A)-dependent HBV antigen presentation. 

It is now unclear if the miRNAs altered in the infected hepatocytes, such as miR-181a and miR-146 [5], that have specific regulatory functions in the immune cells as well [49], could affect directly these cells to support viral evasion. The presence of circulating miRNAs and the existence of intercellular nanovesicle-mediated miRNA transfer that modulates the environment, could potentially support that hypothesis [52,53,54,55,56,57].

### 3.4. Role of miRNAs in the Establishment of HBV Chronic Infection

The natural history of HBV infection shows often a transition from acute to chronic infection, especially in young children. The virus reaches a “dormant” state into the infected hepatocytes, under the cccDNA form, and survive until its eventual life cycle reactivation [3,35,45,58]. One study reported the CpG islands methylation of the cccDNA by DNA methyltransferase 1 (DNMT1) to prevent the viral gene expression and therefore the viral antigen presentation. The DNMT1 overexpression is induced by a decrease of miR-152, under the effect of HBx [35]. 

miR-1 illustrates the duality of actions that can be observed in the course of HBV infection. As said previously, this miRNA can promote viral replication but it can also inhibit the cell proliferation and even induce a reverse cancer cell phenotype [28]. The effect on HCC was confirmed in another study [59]. 

Also, miR-122 can bind directly to the polymerase region in order to repress its expression [37]. Similar observations were made for miR-125a-5p, miR-199a-3p that can affect the S region and miR-210 that can affect the pre-S1 region [38,39]. Since the RNA intermediates of HBV (pgRNA and transcripts) are good targets of miRNA action, it is not surprising to observe several cellular miRNAs targeting them. However, it remains to be determined whether the targeting of HBV transcripts represents an active anti-viral mechanism of the host or if the virus has evolved to hijack these cellular miRNAs in order to reach its “dormant” state.

## 4. Role of miRNAs in HBV-Related Diseases

The modifications induced as a result of HBV infection profoundly alter the cellular and overall organism homeostasis. They are usually associated with diseases, including liver cirrhosis with fibrosis and HCC. The liver cirrhosis turns most of the time into HCC.

### 4.1. Role of miRNAs in HBV-Related Cirrhosis

Numerous studies have tried to identify and characterize the miRNAs involved in liver cirrhosis and therefore differently expressed during this intermediate phase. Roderburg et al. investigated the role of miRNAs in liver fibrosis on a carbon tetrachloride-induced hepatic fibrogenesis and bile-duct ligation mouse models [34]. They observed a significant down-regulation of all the members of the miR-29 family in the two models. The decreased expression was induced by the transforming growth factor beta (TGF-β), inflammatory signals and the nuclear factor kappa B (NFκB) pathways. miR-29c was also identified in another report focusing on the miRNA expression profile in patients with HCC-positive or HCC-negative chronic hepatitis B and hepatitis C virus [22] (Table 1).

Nevertheless, the global miRNA expression profile analysis of human liver tissues from different inflammation, infection and cancer states are not always consistent. It sometimes revealed a particular profile due to the association of both viral hepatitis and cirrhosis [60] or regarding to the type of viral hepatitis [22] and sometimes showed no difference [61]. Further experiments are therefore required to identify the exact molecular mechanisms implicating miRNAs and viral components in the development of cirrhosis and in the transition from cirrhosis to HCC in the patients with chronic viral hepatitis.

### 4.2. Role of miRNAs in HBV-Related HCC

When the cellular modifications and inflammation are too high and maintained for too long, the liver cirrhosis usually evolves into HCC. 

The miR-17-92 cluster is important in the HBV infection and associated HCC. This polycistron includes six miRNAs (mir-17-5p, miR-18a, miR-19a, miR-19b, miR-20a and miR-92a-1) and its upregulated expression is associated with malignancies [62]. By using human HBV-positive human HCC tissues, hepatoma cell lines and woodchuck hepatitis virus-induced HCC animal model [63], Connolly and colleagues were able to demonstrate the elevated expression of miR-17-92 cluster and its implication in the malignant phenotype [29] (Table 1). The expression could be amplified by c-myc activation [64], under HBx control [65], to contribute to HBV latency state [66]. The consequence is the induction of liver oncogenesis. 

Because of its role in immune response, miR-155 is also implicated in hepatocarcinogenesis. Indeed, its upregulation can lead to prolonged exposure to inflammation, a well-known causal agent to cancers like HCC [67]. Using HCC-induced mouse model, Wang and collaborators have demonstrated the oncogenic role of miR-155 at the early stages of the tumorigenesis [30] (Table 1).

To conclude, the liver-specific miR-122 has been extensively studied in the liver-associated diseases. Its expression is low in HCC tissues, including those with viral chronic hepatitis [6,68] (Table 1). As described in point 3.2, the regulation of miR-122 is very complex and helps either promotion or inhibition of the HBV replication. In HCC cells, the “dormant” state of HBV implicates a replication rate very low or inexistent [69]. The recent data accumulate evidence of miR-122 as a highly potential linker between HBV infection and liver carcinogenesis [6,70]. Because of its characteristics, miR-122 is therefore a target of choice for future clinical applications.

## 5. miRNAs as Molecular Tools Against HBV Infection and HBV-Related Diseases

The significance of miRNAs in viral replication, antiviral immunity and liver carcinogenesis emphasizes their values as diagnostic, prognostic and therapeutic targets for HBV infection and HBV-induced diseases. 

miR-122 and miR-18a are of particular interest for diagnostic and/or prognostic applications. They are both released in the blood and could be used as potential non-invasive biomarkers for HBV-related HCC screening [5,53,54]. Some other reports suggest the use of a miRNA panel in order to improve the specificity of the test [55,56]. In addition with the current routinely used markers such as HBV surface antigen (HBsAg), HBV extracellular antigen (HbeAg) and alanine aminotransferase (ALT), the circulating miRNAs represent a significant clinical value for better evaluation of the HBV-infection status, liver injury and early diagnosis of HCC. 

In the therapeutic perspective, the liver cirrhosis is an event prior to HCC development and being able to interfere with this process would prevent carcinogenesis. For example, a strategy based on administration of miR-29 mimic might prevent liver fibrosis (Section 4.1) [34]. However, the disease is often discovered when hepatocarcinogenesis has already developed and HCC does not always show underlying cirrhosis [71]. Finding therapeutic targets involved in HCC is thus a major issue.

For this purpose, the work of Ura’s group is valuable [22]. They analyzed the livers of HBV and HCV positive patients with HCC to identify the miRNAs that are differentially expressed. Nineteen miRNAs were clearly differentiated between HBV and HCV groups, six specific for HBV and thirteen specific for HCV. Based on the miRNAs profile, they made a pathway analysis of candidate targeted genes and were also able to distinguish the cellular mechanisms altered in HBV or HCV-infected livers. The HBV infection alters mostly the pathways related to signal transduction, inflammation and natural killer toxicity, DNA damage, recombination, and cell death, while HCV infection modifies those involved in immune response involving antigen presentation, cell cycle and cell adhesion. Although very interesting, their results are not consistent with those presented in other reports [60,61] and confirmation of the targets needs to be done before considering their clinical application. 

Finally, technological advances in the delivery of miRNA and RNA interference enable safe and efficient *in vivo* miRNA gene therapy, as exemplify by the recent study from Kota and colleagues on the liver cancer [72]. They used an adeno-associated virus to deliver miR-26a in a mouse model of HCC. This resulted in the successful inhibition of the cancer cell proliferation, induction of the tumor‑specific apoptosis, and protection from disease progression without toxicity.

## 6. Conclusions

miRNAs have emerged as new key players in the control of gene expression in cells. Investigations of their profiling have unveiled specific miRNA dysregulations in tumors and during viral infection. 

The HBV is a widespread pathogen that is implicated in HCC development. Numerous cellular miRNAs interacting with HBV have been identified. They reflect the cellular pathways that are altered as a result of the viral infection, viral infection that triggers the liver cirrhosis and carcinogenesis as side effects. On the viral point of view, the dysregulated pathways mirror the strategies of the virus to allow its replication and evade the host defense mechanisms to survive. On the cellular point of view, they mirror the immune response that tries to get rid of the intruder and that becomes dysregulated. The present and future knowledge about the interaction between miRNA, HBV infection and HCC development and progress will probably allow developing strategies and tools to cope, efficiently and at various steps, with the liver carcinogenesis induced by HBV infection. 
